# Influence of individual *HXT *transporters in xylose fermentation by recombinant *Saccharomyces cerevisiae *strains

**DOI:** 10.1186/1753-6561-8-S4-P209

**Published:** 2014-10-01

**Authors:** Davi Ludvig Gonçalves, Akinori Matsushika, Belisa Bordin de Sales, Margareth Patiño Lagos, Tetsuya Goshima, Boris Stambuk

**Affiliations:** 1 Departamento de Bioquímica, Centro de Ciências Biológicas, Universidade Federal de Santa Catarina, Florianópolis, SC, Brazil; 2 Biomass Technology Research Center (BTRC), National Institute of Advanced Industrial Science and Technology (AIST), 3-11-32 Kagamiyama, Higashi-hiroshima, Hiroshima 739-0046, Japan

## Background

Lignocellulosic biomass is an attractive raw material for bioethanol production since it is an abundant and renewable feedstock that does not compete with food and feed production [1]. Xylose is the most abundant pentose present on these feedstocks, and although *S. cerevisiae *cannot readily ferment this sugar, the overexpression of the genes for xylose reductase (XR) and xylitol dehidrogenase (XDH) from *P. stiptis *and xylulokinase (XK) from *S. cerevisiae *allows the utilization of xylose [2]. However *S. cerevisiae *also lacks specific transporters for this sugar and thus the uptake of xylose is carried out by native hexose transporters encoded by the *HXT1*-*HXT7 *genes [3]. In the present report we analyzed the impact of individual *HXT *transporters on xylose fermentation by recombinant *S. cerevisiae *yeast strains overexpressing the genes for XR, XDH and XK [4].

## Methods

Cultivations were perfomed in rich (YP) or synthetic complete (SC) medium containing the required sugars and when necessary, 2% Bacto agar, 0.5 mg/l aureobasidin A and 200 mg/l Geneticin were added to the medium. The chromosome-integrative plasmid pAUR-XKXDHXR [4] containing PGK promoters for overexpression of XR, XDH and XK was digested with *Bsi*WI and then chromosomally integrated into the *AUR1 *locus of the yeast strains. *HXT1, HXT2, HXT5 *and *HXT7 *genes were obtained by PCR from S288c *S. cerevisiae *genomic DNA and cloned individually into a pPGK multicopy plasmid [5], and these plasmids were transformed into the strains lacking all *HXT *genes or individual *HXT *genes, respectively. Anaerobic batch fermentations were performed at 30°C in closed 50-ml bottles with a magnetic stir bar and 100 rpm. Assays with 2-6% of glucose, xylose or both sugars were performed. During fermentation cell growth was monitored and samples were removed for further analysis. Glucose, xylose, ethanol, xylitol, glycerol, and acetic acid were determined by HPLC as previously described [4].

## Results and conclusion

The deletion of individual *HXT *genes had no detectable effect on glucose fermentations, but these knockout strains ferment xylose poorly, even under glucose plus xylose conditions. The low-affinity *HXT1 *permease allowed the maximal consumption of sugars and ethanol production rates during xylose plus glucose co-fermentation, but was incapable to allow xylose consumption when this sugar was the only carbon source. The high-affinity *HXT7*permease allowed efficient xylose fermentation, but during xylose plus glucose co-fermentation this permease showed a clear preference for glucose. While the *HXT5 *permease performed bad with glucose and did not allow xylose utilization, the moderately high-affinity *HXT2 *permease was a transporter that allowed xylose consumption with the same rates as glucose, even under co-fermentation conditions, but had the drawback of producing stuck fermentations. Thus, our results indicate that new approaches to engineer selected *HXT *transporters to increase their affinity towards pentoses, or to avoid their sugar-induced degradation, are promising strategies to improve second generation bioethanol production by xylose-fermenting yeasts.

## Acknowledgements

This work was funded by the Brazilian agencies CNPq, FAPESC and FINEP, and by the Japanese International Cooperation Agency (JICA).
